# How a Latent Virus Eludes Immune Defenses

**DOI:** 10.1371/journal.pbio.0030149

**Published:** 2005-03-22

For a virus to survive, it must elude the ever vigilant immune sentinels of its host. A latent virus can escape immune detection if it resides in nondividing cells and doesn't produce any proteins. No viral proteins means no red flags for immune cells. If the virus targets one of the many cell types that rarely divide, it's relatively safe while latent. But some viruses, like the gamma-herpesvirus, infect B cells of the immune system, which occasionally divide. The gamma-herpesvirus genome persists as circular pieces of DNA called episomes. When an infected B cell divides, the latent gamma-herpes virus episome must replicate and segregate into daughter cells along with the cell's genome. Viral replication and segregation requires the services of a protein called the episome maintenance protein—a potentially recognizable target for immune cells.

Gamma-herpesviruses, including Epstein-Barr virus (EBV) and Kaposi's sarcoma–associated herpesvirus (KSHV), can induce uncontrolled lymphocyte (immune cell) proliferation and result in lymphoma, Hodgkin's disease, and Kaposi's sarcoma. These diseases arise from the persistent latent infections that take hold after initial infections are controlled by immune defenses. The episome maintenance protein produced by EBV, called EBNA-1, harbors an amino acid element in its epitope—the region that binds to a T cell and triggers an immune response—that helps the viral protein evade the killer T cells that could destroy it. Lab studies show that the amino acid element limits EBNA-1's interaction with T cells by inhibiting synthesis and, to a lesser degree, degradation of the protein. How this evasive action works or helps the virus in a living organism is not entirely clear. But if T cells aren't presented with bits of viral protein, they have no way of knowing the virus is present.

In a new study, Neil Bennett, Janet May, and Philip Stevenson explore this question by studying virus–host interactions in mice infected with the murine gamma-herpesvirus-68 (MHV-68). Though MHV-68 infects mice, it behaves similarly to EBV and KSHV infections in humans, producing an acute mononucleosis-like illness and a pervasive pool of latently infected B cells. The episome maintenance protein in MHV-68 and KSHV is called ORF73. None of the viruses can maintain latent infections with deficient episome maintenance proteins.

Stevenson and colleagues first demonstrated that ORF73 limits T cell recognition and then identified a key region responsible for immune evasion by modifying different regions of the viral protein. In the next round of experiments, the authors asked how the viral protein manages this feat. They discovered that ORF73 limits T cell recognition much like EBNA-1 does, by reducing synthesis and degradation of the protein. One region strongly associated with inhibiting epitope presentation to killer T cells corresponded to reduced protein synthesis. When the authors modified the ORF73 transcript to circumvent T cell evasion, the T cells “wiped out” latent virus. These results indicate that avoiding epitope presentation during episome maintenance is key to the virus's survival.[Fig pbio-0030149-g001]

**Figure pbio-0030149-g001:**
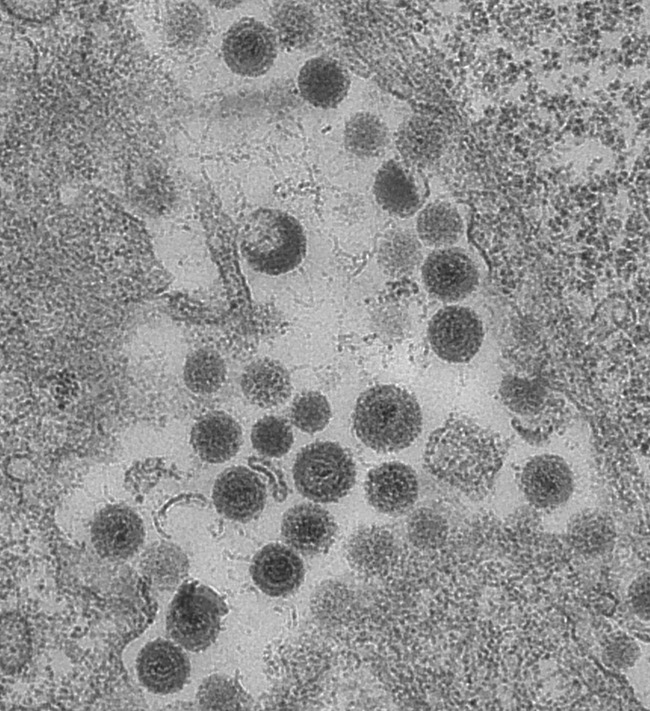
MHV-68 virions emerging from infected cells

Interestingly, the MHV-68 episome maintenance protein mediates immune evasion even though it lacks the amino acid element that does the job for EBV. Future studies will have to determine the responsible MHV-68 epitope and the mechanisms that engineer immune avoidance. Since a majority of epitopes that killer T cells recognize come from aborted translation events, it may be that evasive action is taken at the RNA transcript stage, before RNA is translated into protein. Evading killer T cells, the authors argue, is key to the survival of the gamma-herpesvirus. By figuring out just how evasion occurs, scientists can identify a promising target for controlling infection.

